# Epigenetics modifications and Subclinical Atherosclerosis in Obstructive Sleep Apnea: The EPIOSA study

**DOI:** 10.1186/1471-2466-14-114

**Published:** 2014-07-12

**Authors:** Jose M Marin, Jorge Artal, Teresa Martin, Santiago J Carrizo, Marta Andres, Inmaculada Martin-Burriel, Rosa Bolea, Arianne Sanz, Luis Varona, Javier Godino, Begoña Gallego, Jose A Garcia-Erce, Isabel Villar, Victoria Gil, Marta Forner, Jose P Cubero, Luis Ros

**Affiliations:** 1 Respiratory Service, Hospital Universitario Miguel Servet, I-3 Avda Isabel la Católica, 50006 Zaragoza, Spain; 2 Translational Research Unit, Hospital Universitario Miguel Servet, Zaragoza, Spain; 3 Neurology Unit, Hospital Universitario Miguel Servet, Zaragoza, Spain; 4 Department of Anatomy, Embriology and Genetics, Faculty of Veterinary, Zaragoza, Spain; 5 Cytometry Unit, Instituto Aragonés de Ciencias de la Salud, Zaragoza, Spain; 6 Hematology Service, Hospital San Jorge, Huesca, Spain; 7 Radiology Department, Hospital Universitario Miguel Servet, Zaragoza, Spain

**Keywords:** Sleep apnea, Subclinical atherosclerosis, Systemic inflammation, Epigenetics

## Abstract

**Background:**

Obstructive sleep apnea (OSA) is associated with increased risk for cardiovascular morbidity and mortality. Epidemiological and animal models studies generate hypotheses for innovative strategies in OSA management by interfering intermediates mechanisms associated with cardiovascular complications. We have thus initiated the Epigenetics modification in Obstructive Sleep Apnea (EPIOSA) study (ClinicalTrials.gov identifier: NCT02131610).

**Methods/design:**

EPIOSA is a prospective cohort study aiming to recruit 350 participants of caucasian ethnicity and free of other chronic or inflammatory diseases: 300 patients with prevalent OSA and 50 non-OSA subjects. All of them will be follow-up for at least 5 years. Recruitment and study visits are performed in single University-based sleep clinic using standard operating procedures. At baseline and at each one year follow-up examination, patients are subjected to a core phenotyping protocol. This includes a standardized questionnaire and physical examination to determine incident comorbidities and health resources utilization, with a primary focus on cardiovascular events. Confirmatory outcomes information is requested from patient records and the regional Department of Health Services. Every year, OSA status will be assessed by full sleep study and blood samples will be obtained for immediate standard biochemistry, hematology, inflammatory cytokines and cytometry analysis. For biobanking, aliquots of serum, plasma, urine, mRNA and DNA are also obtained. Bilateral carotid echography will be performed to assess subclinical atherosclerosis and atherosclerosis progression. OSA patients are treated according with national guidelines.

**Discussion:**

EPIOSA will enable the prospective evaluation of inflammatory and epigenetics mechanism involved in cardiovascular complication of treated and non-treated patients with OSA compared with non OSA subjects.

## Background

Obstructive sleep apnea (OSA) is the most common respiratory sleep disorder characterized by recurrent episodes of partial or complete pharyngeal obstruction [1]. Though obesity is the main risk to develop OSA, craniofacial morphology and ventilator drive abnormalities are also important in OSA pathogenesis. Collapse of the upper airway during sleep leads to recurrent arousals, intermittent hypoxia, and surges in sympathetic activity. These intermediate mechanics may explain to some extent, the increased risk of mortality [2], incident hypertension [3,4], coronary artery disease [5] and stroke [6] described in OSA. In this context, circulating biomarkers of inflammation and oxidative stress have been described to be elevated in some OSA patients, and lowered with CPAP therapy regardless of weight changes [7]. A cause-effect relationship has been suggested between this systemic inflammatory state and cardiovascular disease, based on the induction of inflammatory processes in the vessel wall [8]. Such processes are considered to be critical determinants of pathological alterations of the vasculature such as thickening of vessel wall, fatty streak formation, or promotion of atherosclerotic plaques. Furthermore, raised levels of proinflammatory cytokines involved in the atherosclerotic process, such as Interleukin-6 (IL-6) and C-reactive protein (CRP), have been reported in OSA. Nevertheless, these cytokines are also increased in obesity [9] and some studies do not show an independent association between OSA and IL-6 and/or CRP levels [10-12]. Other studies carried in obese patients with and without OSA, found that untreated OSA, rather than obesity, is a major determinant of vascular endothelial dysfunction, inflammation, and elevated oxidative stress in obese patients [13].

Among patients with OSA, the systemic inflammatory variability response can be explained by different pattern of epigenetic modifications induced by the apneic episodes and consequently altered expression of genes involved in the atherosclerotic process. Continuous hypoxia can induce hypermethylation of genes involved in cardiovascular diseases [14,15], but the role of intermittent hypoxia -a characteristic feature of OSA- is not so well known. However, increased methylation in the promoter region of the FOXP3 gene has been recently described in children with OSA and systemic inflammation [16]. The FOXP3 gene controls the differentiation of lymphocytes into regulatory T lymphocytes (Treg), a subset of T helper cells that inhibit atherosclerosis by modulating lipoprotein metabolism [17].

Most OSA patients attending sleep clinics have many confounding comorbidities, e.g. smoking and alcohol habits, obesity, dyslipemia, hypertension, etc.…,. These confounding variables are also associated with systemic low-grade inflammation that makes it difficult to determine the independent role of OSA in the pathogenesis of cardiovascular diseases. Furthermore, the onset of epigenetic changes in adults with OSA remains unknown. Our hypothesis is that changes in epigenetic regulation of systemic inflammation and metabolic dysfunction in OSA, are linked to accelerated cardiovascular morbidity. The Epigenetics Status and Subclinical Atherosclerosis in Obstructive Sleep Apnea (EPIOSA) study is a 5-yr longitudinal study with the overall aim to identify epigenetic markers associated to prevalent and progression of subclinical atherosclerosis in individuals with OSA, as well as biomarkers that may serve as surrogate end-points. The present article describes the purpose and design of the study.

## Methods/Design

### Study objective

The EPIOSA study has the following specific aims: 1) Use of questionnaires, sleep studies, carotid ultrasonography and cerebral magnetic resonance imaging (MRI) for the definition of OSA subpopulations and controls among individuals without comorbidities; 2) Identification and definition of the parameters that predict disease progression over 5 yrs of follow-up. For the purposes of the present study, disease progression is defined in terms of changes in sleep disordered breathing variables, symptoms, carotid artery intima-media thickness (IMT) and plaque and new microblebs in MRI; 3) Identification of useful biomarkers for disease progression by measuring known biomarkers that correlate with OSA severity, in blood and urine samples; 4) Identification of novel epigenetic factors and/or biomarkers correlating with poor outcomes in OSA and controls. This will be analyzed by cytometry analysis, DNA methylation in genes subserving inflammatory functions RNA transcriptomics; and 5) assessment of the effect of CPAP (Continuous positive airway pressure) on inflammatory and epigenetic mechanisms and disease progression.

### Study design

EPIOSA is a 5-yr non-interventional longitudinal prospective study being conducted at the Sleep Clinic of the Hospital Universitario Miguel Servet, a large teaching hospital in Zaragoza (Spain). Following a baseline visit, subjects are to be followed-up at 3, and then every year. All subjects will continue to receive their usual care by their primary physician and sleep specialist throughout the study. CPAP treatment will be started according to the Spanish National guidelines, free of cost for the patients [18].

### Subject participation

We have estimated to enroll up to 300 consecutive OSA male patients aged 20–60 yrs, with baseline apnea-hipopnea index ≥ 5 events per hour of sleep (AHI). In addition, we expect to recruit 50 control subjects (AHI < 5) aged 20–60 yrs and matched by body mass index (BMI). The inclusion and exclusion criteria for OSA patients and control subjects are shown in Table 1. All participants will be advised regarding life-style modification, exercise, diet and to avoid smoking or alcohol consumption.

**Table 1:** Selection criteria

***Inclusion criteria***	***Exclusion criteria***
● Males aged 20 – 60 years	● Any history of cigarette/tobacco smoking use
● AHI ≥ 5 events per hour of sleep (OSA group) and AHI < 5 (control group)	● Alcohol abuse
● Willingness to participate in the study and complying with the study by signing a written informed consent	● Arterial blood hypertension (arterial blood pressure: ≥140 mmHg systolic and/or ≥90 mmHg diastolic or taking antihypertensive medication)
	Low high-density lipoprotein (HDL) cholesterol (<40 mg/dL) or high LDL cholesterol (≥130 mg/dL) or taking lower lipid drugs.
● Available for study visits over 5 years	● Fasting plasma glucose >126 mg/dl or taking anti diabetic medications
	● Other metabolic diseases (e.g., hypothyroidism)
	● Present or past history of vascular diseases, including myocardial infarction, angina, coronary artery procedures (coronary artery bypass graft or percutaneous coronary intervention), aneurysm, transient ischemic attack or stroke.
	● Autoimmune diseases
	● Past or present history of malignancies
	● Chronic inflammatory diseases (e.g., Crohn disease)
	● Chronic infectious diseases (e.g., chronic viral hepatitis)
	● Chronic respiratory diseases (e.g., asthma)
	● Morbid obesity (body mass index ≥40 kg/m^2^)
	● Any chronic oral therapy
	● Sleep disorders other than OSA
	● Having undergone upper airway surgery
	● Having previously use CPAP therapy

### Measurements

#### ***Clinical data***

A questionnaire for data collection was developed. This included assessment of socio-demographic data, weight, height, waist, hip and neck circumferences, healthy behaviors, daily exercise routines, smoking history, consumption of alcohol or other toxic habits, previous medical diagnosis, presence of comorbidities, personal and family history of disease, and medication use. Daytime somnolence is assessed with the Epworth test [19]. Blood pressure is measured according to international guidelines by certified nurses blinded to the presence or absence of OSA [20]. Spirometry is performed according to the standard criteria [21].

#### ***Sleep study***

At our clinic, full polysomnography is used for patients believed to suffer from sleep disorders other than or in addition to sleep apnea, which are not to be included in the present study. For the rest of the patients with suspected OSA diagnosis, a type 3 portable sleep monitor will be used. The sleep study includes continuous recording of airflow from a nasal pressure cannula, thoracic-abdominal motion, oxygen saturation, snoring and body position. Results from all sleep studies are analyze using standard criteria by trained personnel that are blind to the present protocol. An apnea is defined as an absence of airflow of 10 seconds or longer; a hypopnea as an airflow reduction (>50%) lasting ≥ 10 seconds with a decrease in oxygen saturation over 4%. Obstructive apneas are defined as the absence of airflow in the presence of thoracic-abdominal motion. The AHI is calculated based on the average number of apnea plus hypopnea episodes per hour of recording time. Optimal titration of CPAP is obtained by using auto-CPAP (Autoset-T; ResMed, Sydney, Australia), according to previous validation procedures by the Spanish Sleep and Breathing Group [22]. Compliance with CPAP will be measured using the machines’ internal timers. All patients will attend a new sleep study every year.

#### ***Laboratory analysis***

At baseline and every year, whole blood samples are drawn using a 21G butterfly needle into serum gel, EDTA, sodium fluoride and PaxgeneW tubes (PreAnalytix GmbH, Switzerland). Serum glucose, triglycerides, total cholesterol and HDL cholesterol will be measured by spectrophotometry (Chemical Analyzer ILAB 650, Instrumentation Laboratory), and serum apolipoproteins A and B, will be measured by kinetic nephelometry (Immunochemistry Analyzer IMMAGE 800, BeckmanCoulter). Within two hours after collection, high sensitive C-reactive protein (CRP) will be measured based on a particle-enhanced turbidimetric immunoassay technique. For biobanking, about 15 ml of EDTA blood and 5 ml of blood without anticoagulant for serum preparation are to be fractionated into plasma, serum and whole blood and in 1 ml dot-coded metal-free cryotubes are to be stored at -80°C. Inflammatory markers to be assessed are shown in Table 2.

**Table 2:** Outcome measurements in the Epigenetics in Obstructive Sleep Apnea (EPIOSA) study

**Measurement**	**Description**	**Frequency of assessment**
Questionnaires, Dietary, Epworth Sleepiness Scale		Each visit
Vital signs	Including sitting blood pressure, heart rate, height and weight	Each visit
Exhaled carbon monoxide	Confirmation of smoking status	Each visit
Spirometry	Post-bronchodilator FVC, FEV1 and FEV1/FVC	Baseline
Electrocardiogram	12-lead resting electrocardiogram	Baseline
Sleep Study	At home type 3 monitor for diagnosis and at home autoCPAP for CPAP titration (if appropriated)	Baseline and every year
Blood sample	Standard biochemistry and lipid markers: Total cholesterol, LDL, HDL, TG, Apo A, Apo B.	Baseline and last visit
	Inflammatory markers: TNF-α, IFN-ϒ, IL-1β, IL-6, IL-8, IL-10.	
Urine sample	Microalbuminuria, 8-isoprostane	Baseline and every year
Carotid Ultrasonography	High-resolution B-mode assessment of carotid artery intima-media thickness and plaque	Baseline and every year
Magnetic resonance imaging	Cerebral microbleeds detection	Baseline and last visit
Flow cytometry	Fresh peripheral blood T-cell phenotype	Baseline and last visit
DNA methylation	Genome-wide DNA methylation status	Baseline and last visit
miRNA microarray analysis	Levels of candidate miRNAs regulated by aberrant DNA methylation	Baseline and last visit

#### ***Flow cytometry***

At baseline, whole fresh blood with EDTA will be obtained for flow cytometry. Table 3 shows the cells stained, and also the antibodies and kits used. The samples will be incubated for 15 min at room temperature, then acquired in a GALLIOS flow cytometer (Beckman Coulter) and analyzed with the KALUZA software (Beckman Coulter).

**Table 3:** Subset of T cell population to be analyzed

**Antibody-kit**	**Clone**	**Fluorescence**	**Catalog number**
CD4-CD8- CD3	CD4:HP2/6, CD8: 143–44, CD3: 33-2A3	CD4: FITC CD8: PE CD3: PERCP	4FI8PEI3PPI-50 T. IMMUNOSTEP
CD11c	BU-15	FITC	11CF3-100 T IMMUNOSTEP
CD14	47-3D6	APC	14A-100 T IMMUNOSTEP
CD15	MCS-1	FITC	15 F-100 T IMMUNOSTEP
CD16	3G8	PE	16PE2-100 T IMMUNOSTEP
CD45	HL30	CF BLUE	45CFB2-100 T IMMUNOSTEP
CD45 RA	HI100	APC	45RAA2-100 T IMMUNOSTEP
CD 62 L	DREG 56	APC-eFluor 780	47-0629 eBioscience
Human regulatory T cell cocktail*	CD4: SK3, CD25: 2A3, CD127: HIL-7R-M21	CD4: fitc, CD 25: PE-Cy7, CD127: Alexa fluor 647	560249 (BD Pharmigen)

*T reg was defined as CD4+/CD25+/CD127 (low/-) phenotype (ref. [23]).

#### ***DNA methylation analysis***

Part of the blood samples collected in EDTA tubes are stored at -80°C until DNA isolation, which is performed in the Department of Molecular Biology at the Faculty of Veterinary of the University of Zaragoza. Genomic DNA is obtained with the use of the illustra blood genomic Prep Midi Flow Kit (GE Healthcare, UK) following the manufacturer’s instructions. Genome-wide DNA methylation profiles will be studied in approximately 1 μg of genomic DNA treated with sodium bisulfite. The converted DNA will be analyzed using Illumina’s Infinium Human Methylation 450 BeadChip assay (Illumina, San Diego, CA, USA). To minimize position effects, cases and controls will be randomly distributed. The assay allows determination of DNA methylation levels at >450,000 CpG sites covering all designable RefSeq genes, including promoter, 5′, and 3′ regions. It captures CpG islands and shores, non-CpG methylated sites, microRNA promoter regions and disease-associated regions identified through genome-wide association studies [24]. Illumina array data will be processed using the Methylation Module of GenomeStudio v1.9 software. To identify differentially methylated regions (DMRs), we will use a 1-kb sliding window and apply Fisher’s method to combine P values for each 1-kb region on the basis of all probes within each window [25]. Significant DMRs will be selected at a 1% FDR on autosomes and 5% FDR on chrX. After statistical analyses, the methylation patterns of the gene of interest will be confirmed by sequencing of the 24 bisulfite-treated DNA samples used for the genome wide analysis.

#### ***Gene expression analysis***

Blood samples stored at -80°C in PaxGene (TM) tubes will be used for total RNA isolation using PAXgene Blood RNA Kit (Qiagen, Venlo, Netherlands). Quantitative real time polymerase chain reactions (qRT-PCR) will be performed to test whether differential methylation patterns of the selected genes affect their expression level. Complementary DNA will be synthesized from total RNA using random hexamers and the SuperScript^®^ III First Strand Synthesis Kit (Life Technologies, Carlsbad, California, US). Primers and TaqMan^®^ probes will be selected from the Applied Biosystems Inventories Assays and qRT-PCR will be performed in a The StepOne™ Real-Time PCR System (Applied Biosystems, Life Technologies, Carlsbad, California, US).

#### ***MicroRNA analysis***

This study is based in the expression of 96 miRNAs. These miRNAs have been carefully selected based on those predicted by bioinformatic algorithms and databases to regulate genes known to be relevant to inflammation. Total RNA will be extracted from cryopreserved samples using Paxgene Blood miRNA kit (PreAnalitiX) according to the manufacturer’s protocol. RNA integrity will be tested using the 2100 Bioanalyzer (Agilent Technologies, Santa Clara, California, US). Samples will be reverse transcribed using the Taqman^®^MicroRNA Reverse Transcription Kit (Applied Biosystems, Life Technologies, Carlsbad, California, US) and mature miRNAs will be quantified by real-time PCR using the Taqman^®^MicroRNA Assays [26]. miRNA expression will be analyzed using the BioMark 96:96 Dynamic Array (Fluidigm Corporation, San Francisco, California, US). Data will be analyzed using the BioMark Real-Time PCR Analysis Software v2.0 (Fluidigm). The Ct value will be used for analysis by the 2^-∆∆Ct^ method [27].

### Subclinical atherosclerosis imaging

#### ***Carotid intima-media thickness (CIMT)***

At baseline and every year, CMIT are to be assessed in all subjects with an ultrasound system (IU22 Philips). Ultrasound images are acquired by linear high frequency 2-dimensional probes following the protocol of the Bioimage Study [28]. Carotid plaque is defined as a focal structure that protrudes into the lumen of the carotid artery at least 0.5 mm or ≥50% thicker than the surrounding. Three measurements each are made on the far wall of the left and right common carotid arteries in end diastole, 1 cm below the point of bifurcation. The average of the 6 readings will be taken as the CIMT value.

#### ***Magnetic resonance imaging (MRI)***

At baseline and after the 5-year follow up, all subjects are to undergo a cerebral MRI. We use a multisequence MRI protocol on a 1.5-T scanner (GE Healthcare). For cerebral microbleeds detection we will use an accelerated three-dimensional T2*-weighted gradient-recalled echo sequence. MRI scans are viewed by radiology physicians blinded to the OSA status of the subject. As previously described cerebral microbleeds are defined as focal areas of very low signal intensity on T2*-weighted imaging without signal abnormalities on other structural sequences [29]. Microbleeds are classified according to cerebral localization (lobar brain sites or infratentorial locations).

### Power considerations

The calculation of EPIOSA’s planned sample size of 300 patients and 50 matched controls, is based on three estimations: a) in pediatric patients, those with OSA (n = 37), showed a reduced Treg population and altered Th1:Th2 balance toward Th1 predominance compared with non-OSA controls (n = 13) [30]; b) differences among DNA methylation in inflammatory genes were observed among OSA patients with high hsCRP (n = 31), compared with normal non-OSA controls (n = 31) [16]; and C) a randomized study showed that effective treatment of OSA with continuous positive airway pressure for 4 months significantly decreases IMT as early signs of atherosclerosis in 24 patients with severe OSA [31]. We expected a loss to follow-up of an average of 2% per year. In this way at the end of the 5-year follow-up we hope to analyze outcomes in 270 patients with OSA and 45 without OSA. This sample size ensures to analyze subgroups of patients according with prescribed treatment for OSA. Specifically, the sample will have 80% power to detect average IMT differences when comparing OSA patient treated and non-treated CPAP. The power is calculated based on a 2 SD with a significance level of 0.0125.

### Statistical analyses

Groupwise comparisons (n >2) will be performed using a nonparametric Kruskal-Wallis test followed by a Mann–Whitney U test as appropriate. Pairwise comparisons were performed using a nonparametric Mann–Whitney test unless otherwise indicated. Chi square tests will be performed for categorical variables. Regression models will be applied to assess the effect of OSA, biomarkers, peripheral lymphocytes and epigenetic changes on CIMT, carotid plaque and cerebral microbleeds. All models will be adjusted for age and BMI, and additionally for covariables that may change the estimated risk beyond 5% Specific statistics procedures will be performed for the arrays as described above. STATA-12 for Windows (STATA CORP, TX, USA) are to be used for all analyses.

## Discussion

The Epigenetics Status and Subclinical Atherosclerosis in Obstructive Sleep Apnea (EPIOSA) study is a clinical based study of patients with OSA and non-OSA, both without other associated comorbid conditions. EPIOSA aims to elucidate mechanisms involved in the development of OSA complications with a focus on OSA-associated subclinical atherosclerosis. To achieve this, standardized phenotyping of OSA is being performed at baseline and at one and 5 years, with imaging techniques and biobanking of serum, plasma, DNA, mRNA and urine. EPIOSA aims to recruit ambulatory patients attending the Sleep Clinic for study of suspected obstructive sleep apnea.

Prevalence of OSA is increasing in industrialized countries. OSA prevalence parallels the “obesity pandemic”. Both disorders share metabolic and cardiovascular complications that are a major public health and financial challenge. Obesity is a well known risk factor for cardiovascular disorders but the role of OSA as an independent contributor of cardiovascular outcomes is more controversial since most patients with severe OSA are obese. To minimized this problem, in this study we will exclude patients with a BMI > 35 kg/m^2^.

Atherosclerosis is considered the principal contributor of cardiovascular disease. Atherosclerotic lesions and atheromas are localized in the arterial intima-media layers due to accumulation of lipids and inflammatory cells. Endothelial dysfunction triggered by oxidized low-density lipoprotein (OxLDL) is the first step in atherosclerosis. Endothelial cells activation results in the expression of adhesion molecules, cytokines and chemokines that attract circulating monocytes and naïve T lymphocytes. These cells migrate across the endothelium and infiltrate the vascular wall. Monocytes then differentiate into macrophages that take OxLDL to transform in “foam cells”. The accumulation and activation of T cells recognizing OxLDL in the intima constitute the adaptive immunity component in the pathogenesis of atherosclerosis and elicit proinflammatory stimuli that further exacerbate and propagate this disease [32]. The importance of adaptive immune activation has been underlined in the Multi-Ethnic Study of Atherosclerosis (MESA). In the MESA study, which included otherwise healthy individuals, higher circulating CD4+ to CD4+ naïve cells ratio were found to be associated with subclinical atherosclerosis as estimated by carotid ultrasonography [33]. Several subsets of T cells are found within the plaque mainly of the CD4+ phenotypes including Th1, Th2, Th17 and regulatory T cells (Treg) subtypes. Th1 is the most abundant T cell in the human plaque and together with Th17 are considered pro-atherogenic T cells. Th2 cells are rarely detected in lesions and have been suggested to be anti-atherogenic. Treg control the Th1/Th2 balance and are considered to be atheroprotective [34]. Epigenetic regulation is being recognized as an important factor in the pathogenesis of atherosclerosis. The forkhead box transcription factor, FOXP3, is considered the master switch in the regulation of Treg development and function [35]. Furthermore, FOXP3+ Tregs inhibit atherosclerosis by modulating lipoprotein metabolism [17]. A dose-dependent increase in the methylation of the Treg-specific demethylated region in FOXP3 was observed in cultures of peripheral blood mononuclear cells in patients with acute coronary syndromes [36], suggesting that epigenetic suppression of FOXP3 might lead to down-regulation of Treg cells, and in turn increase the risk of atherosclerosis.

There are many potential mechanisms linking OSA, endothelial dysfunction, atherosclerosis and cardiovascular diseases. Sleep loss and fragmentation could trigger endothelial dysfunction and immune response but its remains to be demonstrated if this occurs in patients with OSA. Chronic intermittent hypoxia (CHI) is the other hallmark of OSA. CIH can induce endothelial dysfunction independently of respiratory effort in animal models [37]. In patient with OSA, oxygen desaturation index (ODI), a surrogate of CHI severity has been associated with endothelial dysfunction [38]. On the other hand, ODI emerged as an independent predictor of carotid and aortic wall thickness in OSA patients using cardiovascular magnetic resonance study [39]. CPAP treatment of OSA significantly reduces carotid intima-media thickness, supporting the concept that OSA is an independent risk factor for atherosclerosis [31]. There is growing evidence that OSA is associated with increased activation of immune and inflammatory cells including circulating monocytes, platelets and lymphocytes and proinflammatory cytokines such as leukotrienes, interferon-γ, TNF-α and IL-6 have been reported elevated in OSA [8]. CRP levels have been particularly studied with conflicting results [7,40]. The intermediate process for an increased risk of atherosclerosis in OSA could be the presence of this systemic inflammation and immunity activation as a result of the CHI. There is no evidence of genetic alterations in patients with OSA that favor the development of systemic inflammation, changes the adaptive immunity or that specifically encourage the emergence of early atherosclerosis. However, it is likely a different response from the pro-inflammatory genes to CIH. This response depends in part on epigenetic mechanisms that promote or suppress the expression of these genes. Recently in pediatric OSA those children with elevated CRP, were more likely to have increased DNA methylation of the FOXP3 gene [16]. This result in a reduced Treg population and in a swift Th1:Th2 balance towards Th1 predominance [29]. These immune-inflammatory changes favor endothelial dysfunction and a pro-atherogenic state and ultimately an increased risk for cardiovascular diseases. If those mechanisms also play a role in accelerated atherosclerosis in adults with sleep apnea is unknown. In conclusion, the current study is novel in several ways: 1) It is the first study to evaluate the relationship between baseline OSA phenotypes and normal matched controls without comorbidities and subclinical atherosclerosis and 2) it will allow prospective analysis of inflammatory biomarkers, as well as methylation and miRNA abnormalities in non-OSA subjects and patients with treated or non-treated OSA. Our results may provide more information on the association of OSA and cardiovascular diseases and possibly identifying novel targets for therapeutic intervention. It could also provide evidence that CPAP can not only improve daytime symptoms, but also have a positive effect on progression of subclinical atherosclerosis.

### Ethical aspects

The study is being conducted in accordance with the Declaration of Helsinki and good clinical practice guidelines. All participants must provide written informed consent.

The study has been approved by the Regional Institutional Review Board of Aragon (IACS), Spain, project 03/2013. The study has also been registered at ClinicalTrials.gov identifier: NCT01475421.

## Competing interests

The authors declare that they have no competing interests that are relevant to the content of this article.

## Authors’ contributions

JMM, JA, TM, SJC, BG, study concept and design. MA, IM-B, RB, AS, JG, JAG-E, will carried out the molecular genetic studies and the immunoassays. IV will performed the statistical analysis. VG, MF, JPC will collect the data. LR will interpret the MRI data. All authors critically reviewed and approved the final manuscript.

## Pre-publication history

The pre-publication history for this paper can be accessed here:

http://www.biomedcentral.com/1471-2466/14/114/prepub
